# HIV serostatus and disclosure: implications for infant feeding practice in rural south Nyanza, Kenya

**DOI:** 10.1186/1471-2458-14-390

**Published:** 2014-04-23

**Authors:** Maricianah A Onono, Craig R Cohen, Mable Jerop, Elizabeth A Bukusi, Janet M Turan

**Affiliations:** 1 Kenya Medical Research Institute (KEMRI), Kisumu, Kenya; 2 Department of Obstetrics, Gynecology and Reproductive Sciences, University of California San Francisco, San Francisco, CA, USA; 3 Department of Health Care Organization and Policy, School of Public Health, University of Alabama at Birmingham, Birmingham, AL, USA

**Keywords:** Infant feeding choices, Breastfeeding, PMTCT, Disclosure, Mental health, Kenya, HIV

## Abstract

**Background:**

The World Health Organization (WHO) recommends that HIV-infected women practice exclusive breastfeeding (EBF) for the first 6 months postpartum to reduce HIV transmission. The aim of this study was to determine the effects of HIV/AIDS knowledge and other psychosocial factors on EBF practice among pregnant and postpartum women in rural Nyanza, Kenya, an area with a high prevalence of HIV.

**Methods:**

Data on baseline characteristics and knowledge during pregnancy, as well as infant feeding practices 4–8 weeks after the birth were obtained from 281 pregnant women recruited from nine antenatal clinics. Factors examined included: fear of HIV/AIDS stigma, male partner reactions, lack of disclosure to family members, knowledge of prevention of mother-to-child transmission (PMTCT) and mental health. In the analysis, comparisons were made using chi-squared and t-test methods as well as logistic multivariate regression models.

**Results:**

There were high levels of anticipated stigma 171(61.2%), intimate partner violence 57(20.4%) and postpartum depression 29(10.1%) and low levels of disclosure among HIV positive women 30(31.3%). The most significant factors determining EBF practice were hospital delivery (aOR = 2.1 95% CI 1.14-3.95) HIV positive serostatus (aOR 2.5 95% CI 1.23-5.27), and disclosure of HIV-positive serostatus (aOR 2.9 95% CI 1.31-6.79). Postpartum depression and PMTCT knowledge were not associated with EBF (aOR 1.1 95% CI 0.47-2.62 and aOR 1.2 95% CI 0.64-2.24) respectively.

**Conclusions:**

Health care workers and counselors need to receive support in order to improve skills required for diagnosing, monitoring and managing psychosocial aspects of the care of pregnant and HIV positive women including facilitating disclosure to male partners in order to improve both maternal and child health outcomes.

## Background

Breastfeeding involves a considerable risk of HIV transmission but at the same time not breastfeeding represents a considerable risk to infant survival in low-income countries [1]. Women who practice exclusive breastfeeding (EBF) for the first 6 months postpartum are less likely to transmit HIV to their infants than women who practice mixed feeding [2]. The current WHO guidelines on infant feeding practices for HIV-positive women recommend that, in communities where breastfeeding is the norm, women should practice EBF for 6 months followed by introduction of complementary feeding thereafter [3].

EBF is an alien concept in many African cultures [4]. In fact, the WHO notes that the most common form of infant feeding globally is mixed feeding (breastfeeding along with other liquids and/or foods) and this mode of feeding is often continued for up to or beyond 2 years [5]. The prevalence of EBF globally is 39% and is estimated to be 36% in low-income countries [6,7]. Many women (regardless of HIV status) have the perception that their ‘breast milk is not enough’ in terms of quantity and quality [8] and that milk is a ‘drink’ rather than real ‘food’ [9].

Decisions about the mode and duration of infant feeding are complicated in many settings by the fact that adopting “different” infant feeding practices from those commonly seen in the community can result in unwanted disclosure of HIV-status [10,11]. The fear of HIV transmission through breast milk, even in communities that traditionally breastfeed has developed among HIV-positive women and the health care workers who counsel them [12]. While the current WHO guidelines can be considered feasible and practical for resource-limited settings, the question is whether psychosocial factors such as anticipated stigma, disclosure, and mental health status affect the choice of infant feeding mode in high HIV prevalence settings. Until these questions are answered, “caregivers and mothers in low-resource settings will continue to select options that best suit their own cultural, economic, and psychological needs, and science will need to adapt and design strategies to meet their needs, rather than the other way around” [13].

To address these gaps in the literature, we conducted a post-hoc analysis of the effects of HIV knowledge, and HIV status on EBF practices in a cohort of pregnant and recently postpartum women attending antenatal clinics in rural Nyanza Kenya; including those who were HIV-positive, HIV-negative, and those of unknown HIV status. Additionally, we examined psychosocial factors such as fear of stigma, fear of negative male partner reactions, disclosure to family members, and mental health indicators; and explored how these factors may affect infant feeding choices.

## Methods

### Setting

The Maternity in Migori and AIDS Stigma (MAMAS) Study [14] was conducted in 9 government health facilities that were offering prevention of mother-to-child transmission (PMTCT) services and supported by the Family AIDS Care and Education Services (FACES) to provide antiretroviral therapy (ART) services. FACES is a PEPFAR-funded collaboration between the University of California, San Francisco (UCSF) and the Kenya Medical Research Institute (KEMRI) [15]. The health facilities ranged from sub-district hospitals to health centers. All facilities are located in the southern part of Nyanza Province in Kenya, where the main economic activities are agriculture and fishing. The HIV prevalence among women of reproductive age in this region is 16% [16].

The 2007 Kenya National AIDS/STD Control Program (NASCOP) protocol for PMTCT being used at the time of the data collection for this study included voluntary HIV counseling and testing during antenatal care and retesting in late pregnancy, infant feeding counseling, single-dose Nevirapine, lamivudine for one week and Zidovudine twice daily for 6 weeks for the infant, as well as single dose Nevirapine, lamivudine and Zidovudine for one week for the mother. EBF is recommended for mothers who opt to breastfeed for a period of 6 months and formula feeding is only recommended if Acceptable, Feasible, Affordable, Sustainable and Safe (AFASS). Infant HIV testing is recommended at 6 weeks with DNA polymerase chain reaction (PCR) and followed by antibody testing for HIV at 18 months [16].

### Study population

Only pregnant women who 1) were 18 years of age or older, 2) were in the first seven months of their pregnancy, 3) were appearing for their first antenatal care (ANC) visit of their current pregnancy, and 4) did not know their current HIV status (never tested or tested more than 3 months ago) were eligible for inclusion in the prospective study. All pregnant women were approached to participate in the study in order to reduce stigma that might be associated with participation in the research. Women not meeting study entry criteria received routine health care services. One thousand seven hundred and seven pregnant women provided signed informed consent, met the entry criteria, were enrolled into the study, and completed baseline questionnaires. Findings regarding fears of HIV/AIDS stigma and uptake of HIV testing from the baseline data have been published previously [14]. Following the baseline interview and offer of HIV counseling and testing at the first ANC visit, we selected a total of 614 women for follow-up. All HIV positive women (n = 228) and HIV non-testers (refused or no testing service was available) (n = 158) were selected for follow-up, as well as a random sample of HIV negative women (n = 228) from a larger cohort of HIV negative women (n = 1205). Each month a number of HIV negative women were randomly selected for follow up at each site, equal to the number of HIV positive women identified at that site during that period. For the current post-hoc analysis we used data for women who had both antenatal and postnatal data available on PMTCT related knowledge and actual infant feeding practices (n = 281).

### Study procedures

The Committee on Human Research at UCSF and the Ethical Review Committee at KEMRI in Kenya approved this study. Informed consent was obtained from eligible women coming to the ANC clinics. A baseline questionnaire was then administered to assess demographics and women’s perceptions of HIV-related stigma, knowledge of PMTCT, and choice of infant feeding. Data on HIV status were obtained from medical records after the first ANC visit. Clinical and Community Health Assistants (CCHAs, lay health workers based at FACES-supported health facilities [17]) obtained written informed consent prior to the first interview informed the women that a random sub group would be followed up at home or in the health facility in late pregnancy and 4–8 weeks postpartum. Details regarding where the women lived were also collected at this first contact. At 4–8 weeks postpartum (based on estimated delivery date obtained at the first ANC visit) the CCHAs contacted the women for follow up and if a woman did not show up at the facility, a home visit was conducted during which she was invited to participate in a follow-up structured interview. During the postpartum interview we assessed the same factors included in the baseline interview, but in addition we assessed actual practices in terms of breastfeeding, any experiences of HIV/AIDS stigma and discrimination, evidence of intimate partner violence, and disclosure of HIV status.

### Measures

#### HIV testing

HIV testing was offered in accordance with national guidelines for HIV testing in Kenya that involves serial testing using approved rapid tests. Determine® rapid test (Abbott Laboratories, Wiesbade, Germany) was used for screening [18]. All HIV positive test results were confirmed by SD Bioline HIV1/2(Standard Diagnostics Inc., Kyonggi-do, Korea) rapid test [19]. In the event of discrepant test results, a nationally approved tie-breaker kit that is Unigold™ Recombigen® (Trinity Biotech, Wicklow, Ireland) [20] was used to determine the client’s HIV status and the patient also asked to return for another test in 2–4 weeks to determine their true HIV status.

#### EBF practice

Women who had a living baby at the time of the postpartum interview were asked in an open-ended question how they had been feeding the baby during the past week. The answers were then coded by the interviewer as: 1) breastfeeding only, 2) infant formula only, 3) breastfeeding along with other liquids/foods (water, milk, juice, food), 4) other types of liquid/food only, 5) other, or 6) refuse to answer. Women reporting breastfeeding only (1) were considered to be practicing EBF.

#### Stigma and discrimination scales

To measure anticipated stigma at baseline (before HIV testing) we used a 9 item-scale originally developed in Botswana [21]. “Anticipated stigma” refers to the anticipation that one will personally experience specific types of stigma or discrimination if one is found to be HIV-positive and one’s HIV-positive status is disclosed to others. This scale was found to have good reliability in this sample (Cronbach’s alpha = .86) [14]. Variables were also created to examine anticipated stigma by source; we created dichotomous measures of anticipated stigma from the male partner (answered yes to either or both of the male partner items), from family members (answered yes to either or both of the family items), and from others (answered yes to one or more of the items regarding others).

#### Disclosure

Disclosure of HIV-positive status was measured in the follow-up questionnaires. In a question intended to capture both wanted and unwanted disclosure, HIV-positive women were asked, “Who else knows about your HIV-positive status?” with response choices being husband/male partner, member(s) of own family, member(s) of husband's family, friend(s), health worker(s), other people in the community, and other (again multiple responses allowed). In order to use this variable in the analyses including women of all HIV status categories, we created a composite variable with 3 categories: 1) HIV-positive--somebody knows, 2) HIV-positive—nobody knows, and 3) HIV-negative or unknown HIV-status.

#### Knowledge of mother-to-child-transmission of HIV

Women’s knowledge regarding mother-to-child-transmission was assessed using selected indicators from the Behavioral Surveillance Surveys, which are widely used in Africa [22]. In addition, in an open-ended question, women were asked, “What can a pregnant woman do to reduce the risk of transmission of HIV to her unborn child?” Responses were categorized as: take medication while pregnant, medication for baby soon after the birth, EBF, formula feeding, visit a hospital/clinic/trained health worker, giving birth in a hospital, or other (multiple responses allowed). Knowledge of three or more of these PMTCT methods was considered to indicate knowledge of PMTCT.

#### Postnatal depression

We examined maternal mental health status in the postnatal period using Luo and Swahili translations of the 10-item Edinburgh postnatal depression scale [23]. This scale has been validated in non-English speaking communities [24] including sub-Saharan Africa. A score of 13 or more indicated probable major depression [25].

#### Other predictors

We also examined other potential predictors of EBF, including socio-demographic characteristics, delivery at home vs. in a health facility, and the total number of ANC visits attended during pregnancy.

### Data collection

Interviews were conducted between October 2007 and November 2009. Most of the follow-up interviews took place in the mothers’ homes and were conducted by a trained CCHA in the mother’s preferred language. We used personal digital assistants (PDAs) to collect information on HIV knowledge, PMTCT knowledge, perceptions of stigma, early infant-feeding practices and mental health status [26]. The PDAs were programmed with structured questionnaires in Dholuo, Swahili, and English using survey design software QDS (Questionnaire Development System, Nova Research Company). Participants’ responses were entered directly into PDAs by the interviewers. In cases when the PDA was not available due to lack of battery charge, malfunction, or use by another interviewer, paper questionnaires were used as a back up and later entered into a PDA. PDAs used in the study were password-protected and encrypted.

### Data analysis

A prospective study design allowed us to determine whether or not women with knowledge regarding HIV and their HIV status during pregnancy and specifically with knowledge about mother-to-child transmission of HIV were more likely than others to practice EBF at 4–8 weeks postpartum and whether psychosocial factors such as fear of stigma, negative male partner reactions, lack of disclosure to family members, and mental health could also affect EBF practice.

Data were analyzed using Stata version 11.1 (StataCorp, Texas, USA). Socio-demographic characteristics of the sample were described using univariate statistics. Associations between independent variables and feeding practices postpartum were examined using the chi-square test and Fishers exact test for categorical variables and the Student’s t-test for continuous variables. Subsequently multivariate logistic regression, with 95% confidence intervals (CI) for odds ratios (OR), was used to examine the relative importance of variables identified in preliminary bivariate analyses for EBF practice. After conducting unadjusted logistic regression analyses to identify significant associations, we ran a mixed-effects logit model. This multivariable model accounted included variables that were significantly associated with the exclusive breastfeeding practice in bivariable analyses (p < .05), as well as other variables that have been shown to be important for breastfeeding decision-making in the literature. Specifically, we included socio-economic factors including age, educational level, and occupation, as these factors related to women’s empowerment have been shown to be important factors in decision-making regarding breastfeeding practice in Kenya and similar countries. To diagnose multicollinearity; in the regression model we asked Stata to report the variance inflation factors (VIF) for the predictor variables. Because the women in this study were recruited from 8 different health facilities, we accounted for clustering by site in our multivariate analyses, using robust standard errors.

## Results

Data for 281 women was reviewed. 104 (37.0%) were HIV positive, 123 (43.7%) were HIV negative and 54 (19.2%) had unknown HIV status.

### Socio-demographic characteristics

The mean age of women included in the current analyses was 23.8 years (range 18–40 years). The mean months of pregnancy during first ANC visit was 5.1 months (range 1–8 months) and the average number of pregnancies was 3.2 (range 1–10). Majority of the women had primary school education or less 237(84.3%), were currently living with a male partner 247(87.9%) or married 245(87.2%). Of those currently living with a male partner 63(25.5%) had co-wives. The main occupation was farming/agricultural work for both women 110(39.2%) and their male partners 81(32.9%). Table 1 summarizes basic characteristic of the study participants. The distributions of these characteristics did not differ significantly from those in the larger MAMAS cohort [14] [data not shown].

**Table 1:** Baseline characteristics of women included in analyses of infant feeding practices (N = 281)

**Age group**	**N (%)**
<=20	96 (34.16)
21-30	155(55.16)
> = 31	30 (10.68)
**Education**	
Only primary school (elementary) or less	237 (84.34)
Secondary school (high school) or more	44 (15.66)
**Reading literacy**	
Literate	237 (84.34)
Illiterate	44 (15.66)
**Occupation**	
Housework	65(23.13)
Selling things/fish monger	62 (22.06)
Farming/agricultural work/manual labor	110 (39.15)
Other	44(15.66)
**Marital status**	
Not currently married	36 (12.81)
Currently married	245 (87.19)
**HIV status at 1**^**st **^**ANC VISIT**	
HIV positive	104 (37.01)
HIV negative	123 (43.77)
Unknown HIV status	54 (19.22)
**Currently living with male partner**	
Yes	247 (87.90)
No	34 (12.10)
**Male partner has other wives n (%) (n = 247)**	
Yes	63 (25.51)
No	184 (74.49)
**Male partner’s occupation n = 246 (%)**	
Selling things	31 (12.60)
Farming/agricultural work	81 (32.93)
Fishing	38 (15.45)
Manual labor	36 (14.63)
Other type of job	60 (24.39)

### Bivariate analysis: Factors associated with EBF practice

Regardless of HIV status, majority of the women 222(79.0%) said that they had exclusively breastfed their baby during the past week. About one-fifth (n = 56) had practiced mixed feeding and less than 1.0% had used other type of liquid/food only and no breast milk.

In general, socio-demographic factors such as age, educational status, and currently living with male partner were not significantly associated with EBF.

### Psychosocial factors

At baseline, more than half of the women anticipated HIV/AIDS stigma from some source if found to be HIV-positive 171(61.2%) and approximately one-fifth reported intimate partner violence either during pregnancy or postpartum (n = 57). However, neither fear of HIV/AIDS stigma from anyone (OR 1.46 CI 95% 0.91-2.35) nor fears of negative male partner reactions including intimate partner violence (OR 0.91 95% CI 0.43-1.90) were significantly associated with EBF practice either.

### Postpartum depression

The prevalence of postpartum depression in this group was approximately 10.4%(n = 29). HIV-positive status was associated with high levels of probable postpartum depression (p <0.001); however, postpartum depression was not related to EBF practice (OR 0.79 95% CI 0.28-2.17).

### Disclosure

The rate of disclosure among women who were HIV positive was relatively low 30(31.5%). HIV positive status disclosure among HIV positive women postpartum was significantly associated with EBF practice (p = 0.003).

### PMTCT knowledge

Bivariate analysis showed that in the antenatal period prior to the first ANC visit 99% of women had some knowledge about HIV/AIDS and 88% of the women knew that a mother could transmit HIV to her child. However, at baseline (before the first ANC visit) only 3% of women knew of at least 3 PMTCT strategies. Postpartum knowledge (≥3 specific methods) of PMTCT was significantly higher (p = 0.04). Postpartum knowledge of PMTCT strategies was higher among women who were HIV-positive (56.7%) than in HIV-negative women (46.9%) or those who had unknown HIV status (35.0%). However, this increase in general PMTCT knowledge was not significantly associated with EBF practice (OR = 1.28, 95% CI 0.71-2.29).

(Table 2 summarizes the bivariate associations of potential predictors of EBF practice).

**Table 2:** Bivariate associations of potential predictors of EBF practice (N = 279)

	**N**	**EBF N (%)**	**NON EBF N (%)**	**P value**	**Unadjusted OR (95% CI)**
**Socio demographic characteristics**					
**Education**				0.75	1.14(0.49-2.63)
Only primary school (elementary) or less	217	173(79.15)	44(20.28)		
Secondary school (high school) or more	44	36(81.82)	8(18.18)		
**Age group, years**					
≤20	96				
21-30	153	121(79.08)	32(20.29)	0.54	0.81(0.42-1.56)
≥31	30	22(73.33)	8(26.67)	0.29	0.59(0.23-1.55)
**Number of pregnancies**				**0.99**	0.99(0.48-2.08)
1	54	43(79.63)	11(20.37)		
>1	225	179(79.56)	46(20.44)		
**Currently living with male partner**				0.66	1.22(0.48-3.12)
Yes	245	194(79.18)	51(20.82)		
No	34	28(82.35)	6(17.65)		
**Number of ANC visits completed**				0.053	0.95(0.81-1.12)
1 ANC visit	22	14(63.64)	8(36.36)		
≥2 ANC visit	257	208(80.93)	49(19.07)		
**Place of delivery of the baby**				0.03	2.02(1.04-3.93)
Home	174	132(75.86)	42(24.14)		
Health facility	103	89(86.41)	14(13.59)		
**Anticipated HIV/AIDS stigma from partner**				0.57	1.46(0.91-2.35)
Yes	101	75(75.00)	25(25.00)		
No	149	120(81.08)	28(18.93)		
Refuse to answer	31	27(87.10)	4(12.90)		
**Anticipated HIV/AIDS stigma from others**				0.71	1.08(0.69-1.69)
Yes	171	136(79.53)	35(20.47)		
No	81	63(77.78)	18(22.22)		
Refuse to answer	27	23(85.19)	4(14.81)		
**Any intimate partner violence in pregnancy or postpartum**				0.81	0.91(0.43-1.90)
Yes	57	46(80.70)	11(19.30)		
No	222	176(79.28)	46(20.73)		
**Mental health**				0.65	0.79(0.28-2.17)
Depressed (EPDS score > =13	29	24(82.76)	5(17.24)		
Not depressed (EPDS score < 13)	250	198(79.20)	52(20.80)		
**Post partum Knowledge of PMTCT**				0.40	1.28(0.71-2.29)
High Knowledge (> = 3 methods)	136	111(81.62)	25(18.38)		
Low knowledge (<3 methods)	143	111(76.62)	32(22.38)		

### Multivariate logistic regression for effects of selected predictor variables on EBF Practice

The overall adjusted model was significant (R^2^ = 0.0546, P < 0.01). The mean Variance inflation factor (VIF) for selected predictor variables was 1.06.

Women who were HIV positive were more than twice as likely to exclusively breastfeed than those with negative and/or unknown status (aOR = 2.55 95% CI 1.23-5.27). HIV positive women who had disclosed their HIV status were nearly three times more likely to be exclusively breastfeeding than those who were HIV negative or had unknown HIV serostatus (aOR 2.98 95% CI 1.31-6.79). Additionally women who delivered in hospitals were 2.1 times more likely to practice EBF compared to women who delivered at home (aOR 2.12, 95% CI 1.14-3.95). On the contrary knowledge of PMTCT was not significantly associated with EBF in multivariate analyses, (aOR = 1.20, 95% CI 0.64-2.24) and although HIV-positive status was associated with high levels of probable postpartum depression, postpartum depression was not related to EBF (aOR 1.11, 95% CI 0.47-2.62), when we controlled for the other variables in the model, as shown in Table 3.

**Table 3:** Multivariate logistic regression model parameters for the relationship between selected variables with exclusive breastfeeding practice, accounting for clustering

		**Adjusted model**				**Unadjusted model**			
**Variable**	**Number of women in the category (n)**	**Adjusted odds ratio**	**P value**	**95% Confidence interval**		**Unadjusted odds ratio**	**P value**	**95% Confidence Interval**	
**Place of delivery**									
Home^r^	165								
Health facility	100	2.12	0.01	1.14	3.95	2.02	0.03	1.04	3.92
**HIV status and disclosure**									
HIV negative or unknown status^r^	170								
HIV positive nobody knows or	30	1.55	0.43	0.51	4.71	1.66	0.29	0.65	4.26
HIV positive status somebody knows	64	2.98	0.01	1.31	6.79	4.05	0.005	1.53	10.73
**Postpartum mental health**									
Depressed (EPDS score > =13^r^	29								
Not depressed (EPDS score < 13)	236	1.11	0.79	0.47	2.62	0.79	0.65	0.28	2.17
**Post partum Knowledge of PMTCT**									
Low Knowledge (<3 methods)^r^	135								
High Knowledge (> = 3 methods)	130	1.20	0.55	0.64	2.24	1.28	0.40	0.71	2.29

## Discussion

In this study conducted in a high HIV prevalence area in rural Kenya, we found a relatively high rate of self-reported EBF practice at 4–8 weeks after the birth. Despite this high rate, we found important variations in practice related to HIV status and disclosure, as well as to the place where the woman had given birth (home vs. health facility). Although it is encouraging that HIV-positive women were highly likely to exclusively breastfeed, women who had delivered their infants at home and HIV-positive women who had not disclosed their HIV status, and HIV-negative/unknown status women had lower rates of this important practice, recommended for PMTCT and overall infant health.

### PMTCT knowledge and breastfeeding

Regardless of HIV status every woman should have knowledge of PMTCT. Our study found that overall knowledge about PMTCT was low and even after counseling during ANC visit(s) less than half of the women were knowledgeable about specific methods for PMTCT. The low levels of knowledge on PMTCT measured in the postpartum interviews may be attributed to the fact that although over 90% of pregnant women in Kenya have at least one antenatal visit, less than 47% come for the recommended four visits and only around 50% show up for a post partum visit [27]. These findings bring into question the amount and quality of counseling on infant feeding given during the first ANC visit and concur with similar studies done in Cambodia, Burkina Faso, Cameroon and South Africa that highlight deficiencies in PMTCT counseling given by health care workers. These studies note that in most cases health care workers are not trained to keep up with changing PMTCT guidelines, facilities are understaffed, and health workers lack teaching aids to provide proper counseling. Often, counseling is very one-sided with the provider having a monologue with the patient [28]. Despite the fact that most countries acknowledge WHO recommendations and the need for counseling at a national level, there is a huge gap between the recommended and actual PMTCT counseling and the uptake of the information by the mothers [29]. Poor counseling may result in demotivated mothers who feel that the counseling focus is on the baby and not on them and subsequently do not show any behavioral change as regards PMTCT. Health care workers, on the other hand, may be stressed and frustrated that they cannot give effective counseling [30]. These findings underscore the fact that for PMTCT counseling to be effective; health workers will need support to improve their communication and counseling and support skills.

### HIV status and disclosure

One of the most significant factors that determined EBF in our analyses was disclosure of HIV sero-status. In adjusted analyses, women who were HIV positive and had disclosed their status were nearly three times more likely to exclusively breastfeed than women who were HIV negative or did not know their status. Disclosure of HIV status is a complex issue as it bears significant psychosocial, cultural and economic ramifications. For the cohort of women in this study we found high rates of anticipated male partner stigma and fear of negative male partner reactions including intimate partner violence during pregnancy and postpartum, associated with lack of disclosure of HIV-positive status. A systematic review of 17 peer-reviewed publications reported HIV sero-status disclosure rates among women of 16.7% to 86% [31]. With the current infant feeding guidelines, HIV-positive women can avoid unwanted disclosure of HIV status and have an identity separate from their disease [32] and hence avoid the stigma that is associated with the disease or certain disease specific behavior changes required as in old feeding guidelines recommending formula feeding [33]. A uniform message of EBF to all women regardless of HIV status prevents unwanted disclosure of HIV status and may reduce self-recrimination that HIV positive women feel towards themselves [34].

Conversely, disclosure may help a pregnant HIV-positive woman to take up and adhere to the WHO four pronged approach to PMTCT; which includes 1) safer sex negotiation and behavior change, 2) uptake of contraception to avoid future unwanted pregnancies, 3) uptake of PMTCT preventive strategies such as antiretroviral prophylaxis and EBF, and 4) access to HIV care and treatment for HIV-infected women, their infants, and their families [31,34]. Without disclosure it is difficult for the woman to take medication without being noticed and in a bid to keep her disease private, she may opt out of important PMTCT strategies such as hospital delivery, EBF, contraception, etc. Our study demonstrates that disclosure of HIV status to someone was positively associated with EBF practice, as compared to women who did not disclose their positive sero-status to anyone. A previous study done in Tanzania also showed the importance of disclosure in adherence to formula feeding [35].

### Mental health and EBF

Pregnant women who are HIV-positive are particularly prone to psychological distress, even to the extent of suicidal ideation particularly in sub Saharan Africa settings where there is much stigma and discrimination associated with HIV/AIDS. Studies done in Angola and Zambia have established that women who discover their HIV status while pregnant are more likely than others to develop affective disorders, specifically depression and other somatic disorders than those who do not have HIV or those who know of their HIV status before pregnancy [36,37]. Our study found that women who were HIV-positive were more than three times more likely to exhibit postnatal depression than their counterparts. In separate analyses of MAMAS Study data, we found that internalized HIV-related stigma was highly associated with postpartum depression in HIV-positive women [38]. This has important adverse consequences for the postpartum wellbeing of the mother and the infant. Although our study did not show a statistically significant association between EBF practice and postnatal depression, previous studies have shown that emotionally distressed mothers may find it difficult to adhere to advice concerning their own health and that of their baby which may even include advice on breastfeeding leading to decreased EBF and or breastfeeding duration [39]. It is therefore important that health care workers identify signs and symptoms of psychological distress early in pregnancy. Unfortunately in developing countries where HIV is prevalent, there are not enough health care workers let alone mental health practitioners to manage emotional distress in pregnancy and after the birth. This study highlights an important group of women who, at the very least, need to be given priority access to mental health counseling. We advocate that mental health screening and services be integrated into routine PMTCT counseling.

### Limitations

In interpreting the findings of the present study, several limitations must be acknowledged. Participants analyzed in this study may not be representative of the general pregnant and postpartum women in Kenya. The MAMAS Study in which this study was nested had a high loss-to-follow-up rate between the baseline interview in pregnancy and the postpartum interview. However, no important differences were detected between those women who could be located and interviewed postpartum and those lost-to-follow-up [40]. We had a relatively small sample size of pregnant women for the current analyses (n = 281), limiting our statistical power***.*** The relatively short follow-up period of only 4–8 weeks after the birth of the women participating in this study is likely not to be representative of infant feeding practice beyond this period. The Kenya Demographic Health Survey 2008–09 puts the EBF rate at 0–1 months as 52% and only 40% of infants’ breastfed exclusively by 4–5 months [27]. Additionally data on breastfeeding practice was via self-report and may be subject to social desirability bias. The scope of this study in as pertains to mental health is also limited by the small sample size for showing the influence of postnatal depression and other social factors on breastfeeding.

## Conclusions

This is one of the few prospective studies to examine the many potential factors that could influence EBF practice in high HIV prevalence settings, including HIV knowledge, anticipations of HIV/AIDS stigma, and experiences of postpartum women including intimate partner violence, HIV status disclosure, and depression. Whatever the policies for PMTCT may be, and in particular concerning infant breastfeeding best practices, the psychosocial needs of a pregnant HIV positive woman must be met. The high incidence of anticipated stigma, intimate partner violence, postpartum depression, and the low prevalence of disclosure of HIV sero-positivity provide evidence of the need for health care workers and counselors to receive capacity building to improve skills required to empower women to choose the best infant feeding practices in the era of HIV.

## Competing interests

The authors declare that they have no competing interests. MAO’s work on these post-hoc analyses and the manuscript was supported by a grant from the National Institutes of Health, University of California San Francisco-Gladstone Institute of Virology & Immunology Center for AIDS Research, P30 AI27763 and the University of California, Berkeley Fogarty International AIDS Training Program (AITRP). Award Number K01MH081777 from the National Institute of Mental Health (NIMH), National Institutes of Health (NIH), supported the MAMAS Study and JMT’s effort. The content of this paper is solely the responsibility of the authors and does not necessarily represent the official views of the NIH or CFAR.

## Authors’ contributions

MAO coordinated the study, collected the data, wrote the fist draft of the manuscript, JMT conceived of the study, and participated in its design and coordination and helped to draft the manuscript. MJ contributed to subsequent drafts and performed additional statistical analysis, EAB and CRC provided academic guidance and contributed to subsequent drafts. All authors read and approved the final paper.

## Pre-publication history

The pre-publication history for this paper can be accessed here:

http://www.biomedcentral.com/1471-2458/14/390/prepub
